# A Nosocomial Outbreak of Invasive Listeriosis in An Italian Hospital: Epidemiological and Genomic Features

**DOI:** 10.3390/pathogens10050591

**Published:** 2021-05-12

**Authors:** Valeria Russini, Martina Spaziante, Tiziana Zottola, Anna Giovanna Fermani, Gina Di Giampietro, Giovanni Blanco, Paolo Fabietti, Riccardo Marrone, Roberta Parisella, Sergio Parrocchia, Teresa Bossù, Stefano Bilei, Maria Laura De Marchis

**Affiliations:** 1 Istituto Zooprofilattico Sperimentale del Lazio e della Toscana “M. Aleandri”—Sezione di Roma, 00178 Rome, Italy; valeria.russini-esterno@izslt.it (V.R.); gina.digiampietro@izslt.it (G.D.G.); teresa.bossu@izslt.it (T.B.); stefano.bilei@izslt.it (S.B.); 2 Regional Service Surveillance and Control for Infectious Diseases (SERESMI), National Institute for Infectious Diseases “Lazzaro Spallanzani” IRCCS, 00149 Rome, Italy; martina.spaziante@inmi.it; 3 Istituto Zooprofilattico Sperimentale del Lazio e della Toscana “M. Aleandri”—Sezione di Latina, 04100 Latina, Italy; tiziana.zottola@izslt.it; 4 Department of Prevention, Azienda Sanitaria Locale Latina, 04100 Latina, Italy; a.fermani@ausl.latina.it; 5 Ospedale Santa Maria Goretti, 04100 Latina, Italy; g.blanco@ausl.latina.it (G.B.); p.fabietti@ausl.latina.it (P.F.); r.marrone@ausl.latina.it (R.M.); ro.parisella@ausl.latina.it (R.P.); s.parrocchia@ausl.latina.it (S.P.)

**Keywords:** listeriosis, whole genome sequencing, nosocomial outbreak, *Listeria monocytogenes*, ST451

## Abstract

*Listeria monocytogenes* (*L. monocytogenes*) is a widespread opportunistic pathogen that causes the listeriosis foodborne disease. This bacterium has become a common contaminant of handled food, and a relevant public health issue. Here we describe a nosocomial outbreak of listeriosis caused by an ST451 strain of *L. monocytogenes* involving three cancer and one immunocompromised patients hospitalized in different units from the same hospital during September and October 2020. The epidemiological investigation was conducted using traditional microbiological methodology combined with a whole genome sequencing approach. The source of contamination was identified in the kitchen hospital, where a meat slicer used to prepare patients’ meals was tested positive to the same sequence type (ST) of *L. monocytogenes*. This is the first report of an outbreak of listeriosis caused by ST451 in Italy.

## 1. Introduction

The Gram-positive *L. monocytogenes* is a facultative intracellular bacterium responsible for the food-borne disease listeriosis [1,2]. *L. monocytogenes* represents a major threat to the food industry and public health since it can grow at refrigeration temperatures, high salt concentrations, and over a large range of pH [3]. Invasive listeriosis can manifest as septicemia and meningoencephalitis and it is characterized with high mortality rate, especially in the elderly and immunocompromised hosts [2].

The incidence of infection ranges from 0.8 cases per 100,000 in North America to 0.2 cases per 100,000 in Europe, mostly presenting as a sporadic disease, however food-borne outbreaks have also been described [4]. Nosocomial outbreaks of listeriosis have been also reported, especially among newborns and immunocompromised patients [5,6,7]. 

*L. monocytogenes* species is highly heterogeneous: it can be divided into 13 serotypes [8] and four PCR serogroups [9] related to four main lineages [10]. These categories can be further subdivided into geographically and temporally widespread clones by sequencing a small number of housekeeping genes with multilocus sequence typing (MLST) [11]. From an epidemiological point of view, a more accurate identification of molecular clusters deriving from associated food products, environment, and human cases can be achieved by using whole genome sequence (WGS) data. In particular, the core genome MLST (cgMLST)-based typing is one of the most helpful methods for identification of epidemiological clusters [12].

Here, we describe a cluster of four cases in the same hospital in the city of Latina (Lazio, center of Italy) that occurred in September and October 2020, caused by an ST451 strain of *L. monocytogenes*, which resulted to be linked to a contaminated meat slicer.

## 2. Results

### 2.1. Description of the Clinical Cases

**Figure 1:**
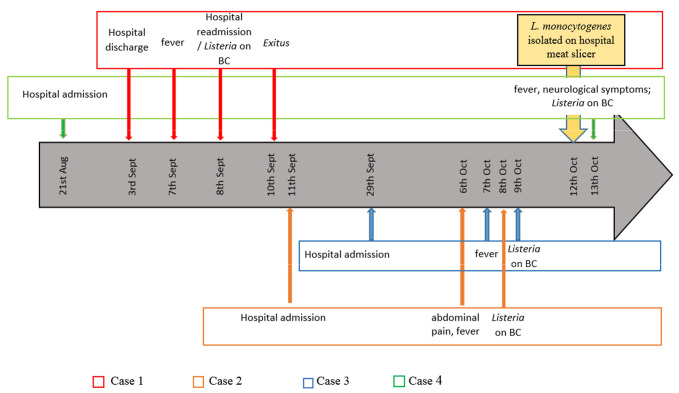
Timeline of epidemiological, environmental, and laboratory investigation of an outbreak of invasive hospital-acquired listeriosis linked to a contaminated meat slicer. BC: blood cultures.

The clinical course of the four human cases is described in detail below and summarized in Figure 1.

#### 2.1.1. Case 1

A 53-year-old man was hospitalized for abdominal pain and diagnosed with metastatic gastric adenocarcinoma. Five days after being discharged from the Oncology Unit, he was readmitted to the hospital due to the occurrence of fever, respiratory distress, and neurological symptoms. At the emergency room, he was diagnosed with a severe bilateral pneumonia with pleural involvement, and he started a treatment with intravenous (IV) meropenem 1 g every (q) 8 h, IV linezolid 600 mg q12 h, IV levofloxacin 750 mg q24 h. IV methylprednisolone (40 mg q12 h) was added in order to improve respiratory distress. *L. monocytogenes* was isolated by blood cultures. The patient died 72 h after the hospital readmission.

#### 2.1.2. Case 2

A 75-year-old man, affected by cholangiocarcinoma, arterial hypertension, and COPD (chronic obstructive pulmonary disease) was admitted to the Geriatric Unit due to abdominal pain and jaundice. A total of 29 days after the hospital admission, due to the occurrence of fever, he underwent blood culture that documented the growth of *L. monocytogenes*. A corticosteroid treatment (oral prednisone 25 mg q24 h) was prescribed for acute COPD exacerbation. Furthermore, according to the patient’s impaired renal clearance, a dose-reduced IV ampicillin (2 g q6 h) and gentamycin (160 mg q24 h) antibiotic treatment regimen was initiated but discontinued after 12 days due to the development of a *Clostridium difficile* infection. After a 10-day treatment regimen with the vancomycin oral antibiotic, the patient recovered and was discharged at home 34 days after the onset of the septic episode.

#### 2.1.3. Case 3

A 55-year-old woman affected by common variable immunodeficiency and autoimmune enteropathy treated with oral corticosteroids was admitted to the Infectious Diseases Unit due to pancytopenia and anasarcatic status. Her hospital diet was formulated according to her gluten sensitivity and lactose intolerance. Eight days after hospitalization, she developed fever and *L. monocytogenes* was isolated from the blood culture. She was treated with a weight-adjusted antibiotic regimen of IV ampicillin 2 g q6 h and IV gentamicin 160 mg q24 h for 14 days with clinical improvement, and was discharged at home 23 days from the growth of *L. monocytogenes* from blood culture.

#### 2.1.4. Case 4

A 39-year-old man was admitted to the Neurosurgery Unit due to the occurrence of headache associated with temporal and spatial disorientation. A recurrence of his recently operated glioblastoma was diagnosed. Steroid treatment (betamethasone 4mg IV q12 h) was initiated in order to reduce cerebral oedema, and he was transferred to the Oncology Unit to undergo radiotherapy. After 23 days from the hospital admission, he developed fever and his neurological status deteriorated. *L. monocytogenes* was isolated by blood cultures the same day, whereas the culture of cerebrospinal fluid did not provide diagnostic information. He recovered from the infection following 21 days on antibiotic treatment with IV ampicillin 2 g q4 h and IV gentamicin 240 mg q24 h and was referred to hospice care.

### 2.2. Epidemiological Investigation Results

The available clinical reports, the epidemiological questionnaires, and menus provided by the hospital dietetics service showed that for the duration of the hospitalization, cases 2 and 4 consumed only hospital meals, while case 3 claimed to have also eaten, on one occasion, a parmesan cheese portion brought from home and stored in a thermic bag for a few days. It was not possible to collect detailed information concerning the meals consumed by case 1. However, the intake of an unrestricted diet in the period preceding the onset of symptoms is reported on his clinical record. Case 2 followed a standard diet. Among the suspected foods that could be related to the transmission of *L. monocytogenes* (i.e., ready to eat) or those possibly manipulated on the meat slicer, we identified fennels, chicken salad, mozzarella cheese, and turkey meat. Having followed a low-residue and gluten-free diet, it was assessed that case 3 consumed neither raw vegetables (only fresh fruits) nor milk and milk derivatives. In the days preceding the onset of the symptoms of listeriosis, case 3 ate roasted pork and raw ham. Case 4 consumed a very varied diet, including different types of cheese (stracchino, certosino, robiola, mozzarella, Emmenthal), raw vegetables (green and mixed salad, fennels, tomatoes, fresh fruits), cooked ham, and roasted meat (turkey and pork).

After the identification of the meat slicer as the contaminant source, a temporary suspension and the adoption of an external service for the supply of packaged meals were ordered to perform an extraordinary sanitation intervention in the hospital kitchen. The kitchen management company subsequently restored the environment in compliance with the parameters of the Reg (EU) 2017/625. Afterwards, swabs on the same surfaces were carried out by another laboratory accredited according to the UNI EN ISO 17025 standard. Having ascertained the absence of *L. monocytogenes*, after five days the cooking center was reopened. No other investigation was carried out by the hospital staff.

### 2.3. Bacterial Identification and Genomic Analysis

After inspection and environmental sampling of the hospital kitchen, the only environmental sample positive for *L. monocytogenes* was a kitchen slicer, declared as exclusively dedicated to cutting raw meat and, after a sanitizing step, cooked meat (e.g., roasts). This isolate was analyzed in parallel with the four human strains obtained from the cases of September and October 2020. Unlike those of human origin, the environmental strain required different subculture steps to allow the characterization of flagellar antigens. All the isolates were assigned to the serotype 1/2a. Antimicrobial susceptibility test (AST) indicated resistance to erythromycin and trimethoprim/sulfamethoxazole for cases 1 and 3, and to trimethoprim/sulfamethoxazole for cases 2 and 4.

Sequencing data confirmed serogroup IIa for the five isolates and MLST analyses indicated that they all belonged to sequence type (ST) 451, clonal complex (CC) 11, lineage II. The identified cgMLST profile comprised a novel allelic combination (CT9151) including twelve new alleles with respect to the reference database of the Pasteur Institute BIGSdb-*Lm* (Paris, France) [13,14]. The cgMLST profile was the same for all the tested samples. With a retrospective approach, we analyzed the sequence data of the available strains isolated from listeriosis cases treated in the same hospital over time (seven cases starting from 2017). The minimum spanning tree showed that only the last four cases clustered with no allelic differences (Figure S1). The SNPs analysis confirmed that the outbreak isolates were closely related, from a minimum of zero to a maximum of six SNPs differences detected in the whole genome (Table S1). Within the outbreak strains, 0–3 SNPs differences were found in the core genome, further confirming the existence of a genomic cluster. All the human isolates considered in the study carried the genes for resistance to the fosfomycin (*fosX*), lincomycin (*lin*), quinolone (*norB*), and sulfonamides (*sul*) antimicrobial.

### 2.4. In-Depth Study of L. monocytogenes ST451 in a National and International Context 

**Figure 2:**
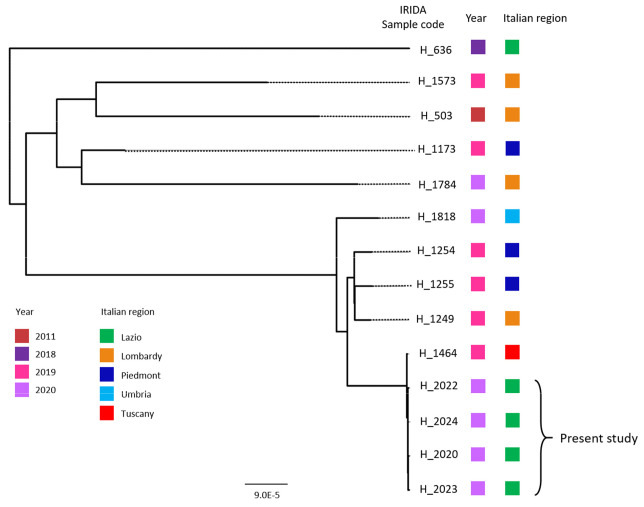
Phylogenetic tree based on SNPs (SNP Observer Pipeline on IRIDA ARIES) of all samples belonging to ST451 in the IRIDA database.

To evaluate the occurrence of the ST451 over Italy, we queried the online platform IRIDA-Aries, whose database, named IRIDA, comprises the sequenced *L. monocytogenes* clinical isolates collected in Italy in recent years. At the moment of submission, a further ten cases of *L. monocytogenes* belonging to ST451 were found on the platform (one from Lazio, one from Tuscany, one from Umbria, four from Lombardy, and three from Piedmont). All the strains were assigned to IRIDA cluster 81, with the exception of samples H_1784 and H_503. The comparative genomic analysis, using the Aries “SNP analysis” tool, showed that the new four isolates of Lazio clustered with a Tuscan case (H_1464), previously collected and sequenced by our laboratory (Figure 2). After contacting the Regional Reference Center for Foodborne disease of the Tuscany region (Centro di Riferimento Regionale sulle Tossinfezioni Alimentari—CeRRTA) it emerged that this strain was isolated from the blood of an 81-year-old woman suffering from oncological disease, whose diet consisted of homogenized or pureed food (personal communication). The woman died later. The strain shared the same cgMLST type with the nosocomial outbreak strains, with one allelic difference, and showed 4–6 SNP differences over the whole genome.

Further investigation was conducted by searching for ST451 *L. monocytogenes* isolates from food samples among those received by our laboratory during routine activities and consulting the Reference National Laboratory for *L. monocytogenes* (personal communication). A single ST451 case was found in the records of our laboratory and concerned four bacterial strains isolated from a pecorino cheese produced in 2019 from a Tuscan farm. Following the workflow described in materials and methods, the food isolates compared to the human ones showed an average of 22 allelic differences (min 20, max 25) in the core genome analysis (Figure S1), and belonging to a different cgMLST profile (CT9149). The four strains from cheese differ from those of the nosocomial outbreak by 284 to 287 (mean 285.2) SNP differences. Among these, 25 were located in the core genome of *L. monocytogenes*. Considering the estimated mutation rate of the core genome of 0.41 SNPs per year [12], the outbreak cases were separated from the cheese isolates around 60 years ago, thus no link was proven. To confirm this, we interviewed the manager of the producing farm to evaluate the possibility of epidemiological links with the hospital food suppliers, but no evidence of a relationship was found.

A total of 90 out of 92 virulence genes was identified in all the ST451 strains available (nosocomial outbreak strains, pecorino cheese strains, and the Tuscan listeriosis case strain) (Table S2). The samples shared the same allele with the main virulence genes, with the notable exception of the gene of internalin A (*inlA)*, that mediates the entry of *L. monocytogenes* into intestinal epithelial cells of the host [11]. In this case, the nosocomial outbreak strains and the Tuscan strain did not match any existing allele in BIGSdb-*Lm* database [14], and showed a mutation in position 2152 of allele 64 (C->T) that causes a missense mutation (proline to serine).

Further investigation was conducted by searching for ST451 *L. monocytogenes* isolates in public databases. A total of 47 samples were found over 2010–2019. The samples were from Poland (five human and one food strains), Switzerland (four environmental strains), Austria (twelve human, two food, and eleven unknown strains), and USA (three human, one food, two environmental, and six unknown strains) (Table S3). The cgMLST were identified for each sample and the minimum spanning tree of allelic profiles showed that the nosocomial outbreak did not cluster with any of other strains among those evaluated (Figure S2).

## 3. Discussion

Whereas a direct or indirect person-to-person transmission was suggested in some *L. monocytogenes* nosocomial outbreaks, especially among neonates [5,15,16], the majority of transmissions turned out to be linked to contaminated food products such as vegetables [17], sandwiches [18], dairy products such as pasteurized milk cheese [19], ice cream [20], or milkshakes [21], meat jelly [22], or other meat products [23]. In particular, in 2015 in a Washington State hospital, a case of hospital-acquired listeriosis resulted to be closely related to two previous cases that occurred in the same hospital one year before, and linked to a milkshake machine persistently contaminated despite repeated cleaning and sanitation [24].

We described a small nosocomial outbreak of invasive listeriosis linked to a contaminated meat slicer employed in a hospital kitchen. From the available menus of the affected patients, it was not possible to identify one food product as the common direct source of infection. Our findings therefore support the hypothesis of a secondary contamination event involving a food treated with the slicer. We cannot rule out the intake of cross-contaminated food by other kitchenware or surfaces, not sampled during the inspection, or above which the contamination did not last over time. The original source of the kitchen contamination, by this peculiar strain of *L. monocytogenes*, is yet to be identified.

*L. monocytogenes* is able to tolerate adverse environmental conditions by forming biofilm and by developing stress-resistant mechanisms, surviving for long periods on food processing plants [25]. On stainless steel, this microorganism may produce extensive biofilms and an extracellular matrix that, along with the conformation of some food processing plants (e.g., presence of reservoirs, mixers, and pipes more tough to clean), could explain why *L. monocytogenes* may persist in the environment and is difficult to sample [26,27]. In our case, the environmental isolate still retained the ability to grow in culture even though several subculture steps were necessary in order to serologically characterize the flagellar antigens.

In our study, we provided genomic characterizations of strains involved in an outbreak, together with the analysis of virulence genes profile including all the strains of ST451 isolated from food available to date in Italy (Table S2). In particular, we identified a missense mutation in the *inlA* gene, which was not previously described and was specifically associated to the outbreak related cases. Internalin (*inlA*) is a surface protein that mediates the entry of the pathogen into various non-phagocytic human eukaryotic cells [28,29,30,31] and plays a key role in the crossing of the intestinal barrier, allowing the bacterium to reach the host bloodstream [32]. Given these premises, it would be interesting to study the effects of this mutation on the in vitro and in vivo activity of the protein.

To our knowledge, this is the first case of an outbreak linked to ST451 *L. monocytogenes* described in Italy, whereas several cases of ST451 were reported in other European countries. In literature are reported strains isolated from two patients in France in 2017, four patients in Germany in 2015–16, and ten patients in Poland in 1997–2013 [12,33,34], in samples from meat [35] and farm environment, as well as fecal isolates from ruminants in Switzerland [36]. Finally, from a surveillance study conducted in Austria for the year 2017, it emerged that the ST451 was the third most common ST among the collected human isolates, causing three cases and accounting for 5% of the food isolates (especially dairy products) [37]. The comparative analysis of the available sequences from European and American ST451 strains did not show any correlation with the outbreak here described (Figure S2). We must consider that available data are incomplete and non-representative since they were published only from a few countries (Table S3). Furthermore, the comparison with data relating to the non-human ST451 clusters so far identified did not provide any backtrack information on the food products liable for onset of Italian cases. Given the geographical proximity, it could be interesting in the future to extend the analysis to human isolates collected in Northern Italy.

Listeriosis may represent a life-threatening disease for vulnerable, hospitalized, and immunocompromised patients, as well as the elderly, newborn infants, and pregnant women, being at high risk of developing invasive disease. In fact, in our small case series, three patients were diagnosed with cancer and one was affected by common variable immunodeficiency. We may also hypothesize that other patients consumed contaminated food but developed only mild, gastrointestinal symptoms not leading to a diagnostic work-up.

Under these premises, healthcare professionals should counsel people at increased risk for severe listeriosis to avoid foods that may be more likely to be contaminated by *L. monocytogenes*, such as lightly cooked or raw ready-to-eat products. Hospitals and healthcare facilities should be aware of the risk of *L. monocytogenes* contamination of food service equipment and food processing plants as well.

In the reported cases, the constant surveillance activity of the CC-ICA (Comitato di Controllo delle Infezioni correlate all’assistenza—Committee for the Control of Care-Related Infections) and an intense collaboration with the territorial public hygiene services allowed stopping the epidemic cluster and interrupted the spread of the infection originating from *L. monocytogenes*.

This study emphasizes the importance of a systematic and real-time whole genome sequencing of isolates of human, food, and environmental origin, according to a one-health approach, to monitor the development of an epidemic cluster and to quickly identify the cause of the pathogen spread.

Furthermore, as in our case, surveillance strategies involving a close collaboration between diagnostic laboratories, clinical departments, epidemiological observatory, and public health local services is pivotal in order to early identify outbreaks and prevent their further spreading.

## 4. Materials and Methods

### 4.1. Outbreak Investigation and Sampling

Due to this unusual recurrence of infections, the infectious disease consultant reported the cases to the Health Director of the hospital. A collaboration was activated with the restricted CC-ICA group (Comitato Controllo delle Infezioni correlate all’assistenza—Committee for the Control of Care-Related Infections) and the SIAN (Servizio Igiene degli Alimenti e Nutrizione—Food Hygiene and Nutrition Service) with which all the subsequent initiatives were settled. The investigation of the outbreak was conducted by the Prevention Department of the Local Health Authority, starting from inspection and environmental sampling of the hospital kitchen. Sampling was performed by means of sterile swabs, sponges, and containers. In particular, a sanitized lunch tray (100 cm^2^ surface), and not-sanitized internal shelf of the blast chiller (100 cm^2^ surface), a sanitized vegetable cutting board (100 cm^2^ surface), sterile gloves of the kitchen operators, a sanitized thermal sealer for single portion packaging, and a meat slicer, working at the moment of inspection, were sampled.

The hospital staff collected, when possible, directly from the patient, the epidemiological questionnaires used in the survey on potential food exposures during the 30 days before illness onset. Moreover, a detailed dietary history of the affected patients was collected from the dietetics service report.

### 4.2. Microbiological Methods for Bacterial Identification and Characterization

In the hospital microbiology laboratory, isolation of *L. monocytogenes* from human cases was carried out by means of blood culture bottles, Columbia agar +5% sheep blood, and Columbia CNA agar +5% sheep blood (bioMérieux SA, Marcy-l’Etoile, France). *L. monocytogenes* identification was confirmed by MALDI-TOF VITEK MS system (bioMérieux SA, Marcy-l’Etoile, France) and microscopically, by Gram staining procedure using Color Gram 2 (bioMérieux SA, Marcy-l’Etoile, France).

The analysis of the environmental samples was carried out at the food microbiology laboratory of Istituto Zooprofilattico Sperimentale del Lazio e della Toscana (IZSLT)—provincial division of Latina. The detection of *L. monocytogenes* was performed using VIDAS LMO2 kit (bioMérieux SA, Marcy-l’Etoile, France) by enzyme-linked fluorescent assay (ELFA) method. Positive samples were cultured according to UNI EN ISO 11290-1:2017, using ALOA (agar *Listeria* according to Ottaviani and Agosti) medium and LSM (lymphocyte separation medium) as the second selective medium. The biochemical culture confirmation was carried out performing beta-hemolysis test, L-rhamnose test, and D-xylose test.

The environmental isolate from the meat slicer and the clinical isolates of *L. monocytogenes*, obtained from blood culture at the hospital microbiology laboratory, were transferred to the laboratory of the Regional Reference Centre for Pathogenic Enterobacteria (CREP) at the Food Microbiology Unit of IZSLT—central division of Rome. The samples were serotyped by using the Mast assure antiserum *Listeria* “h” and “o” (Mast Group Ltd., Liverpool, UK) according to manufacturer instructions.

The antimicrobial susceptibility test (AST) was carried out using the Kirby–Bauer disk diffusion susceptibility test on Mueller–Hinton agar medium.

### 4.3. Whole Genome Sequencing of Bacterial Isolates

Genomic DNA was extracted with the automatic extraction system QIASYMPHONY (QIAGEN). Libraries were prepared using Nextera XT DNA Library Prep and run with a MiSeq sequencer (Illumina) pair-end (2 × 300 bp).

Raw reads were submitted to the Sequence Read Archive (SRA) at GenBank database of NCBI and are available under the bioproject PRJNA708157, biosamples SAMN18220286 (Case 1), SAMN18220287 (Case 2), SAMN18220288 (Case 3), SAMN18220289 (Case 4), SAMN18220290 (meat slicer), SAMN18631085 (Case from Tuscany), SAMN18631086 (Pecorino cheese strain 2), SAMN18631087 (Pecorino cheese strain 3), SAMN18631088 (Pecorino cheese strain 4), and SAMN18631089 (Pecorino cheese strain 5).

Raw reads quality was assessed with Fast QC v0.11.5 [38] and low-quality sequences and adapters were trimmed using Trimmomatic v0.36 [39]. The following quality filters were used: minimum quality of Q30 to keep a base from the beginning and from the end of the read, a window size of 10 with Q20 as average quality, and a minimum length read of 50 bp. The high quality reads were de novo assembled into contigs using SPAdes v3.9.1 with the “careful” option [40], and the draft assemblies were improved using Pilon v1.23 [41]. The resulted assemblies quality were assessed with QUAST v5.0.2 [42]. The serogroup was in silico deduced with BLAST v2.10.1 [43] by evaluating the composition of selected loci [9]: *lmo0737*, *lmo1118*, *ORF2110*, *ORF2819*, and *prs* (*lmo0199*). We analyzed the seven housekeeping genes (*abcZ*, *blgA*, *cat*, *dapE*, *dat*, *ldh*, and *lhkA*) identified by the Pasteur Institute BIGSdb-*Lm* schemes to perform in silico subtyping by using the tool MLST (Github https://github.com/tseemann/mlst, accessed on 1 March 2021) [13] for all the sequenced genomes. The cgMLST scheme consists of 1748 highly conserved core genes from the *L. monocytogenes* EGD-e reference strain. This genotyping method defines cgMLST types (CTs) as groups of cgMLST profiles th at differ by up to seven allelic mismatches out of 1748 loci [13,14,44]. The cgMLST analysis was performed using chewBBACA v2.7 [45] against the *Listeria* cgMLST database [14]. The new cgMLST profiles of the tested samples were assigned by the Pasteur Institute BIGSdb-*Lm*. Minimum spanning trees of the allelic results were generated using the MSTreeV2 algorithm in GrapeTree software [46] using other *L. monocytogenes* cases that occurred in the past years in the same geographic area.

The SNPs analysis was performed with the pipeline CSI phylogeny v1.4, attainable from the Center for Genomic Epidemiology (www.genomicepidemiology.org, accessed on 1 March 2021) [47], using both whole genome and core genome. The SNPs were filtered according to parameters: a minimum distance of 10 bps between each SNP, a minimum of 10x depth and 10% of the breadth coverage, the mapping quality was above 30, and the SNP quality was higher than 25.

Identification of acquired antibiotic resistance genes were assessed from genome assemblies by using the BLASTN algorithm [43] implemented in the BIGSdb-*Lm* platform [13,14] The samples were tested for the main virulence genes using the software VirulenceFinder 2.0 [43,48,49] with the database curated by Dr. Flemming Scheutz (Statens Serum Institut, Denmark).

The comparative genomic analysis of the Italian human strains was conducted with the online platform Integrated Rapid Infectious Disease Analysis (IRIDA) Advanced Research Infrastructure for Experimentation in Genomics (ARIES) maintained at the ISS (Istituto Superiore di Sanità, Italian National Institute of Health) [50,51]. The SNP-based phylogenetic analysis was carried out using PopPUNK tool [52] (SNP Observer Pipeline), which applies neighbor joining on the distance matrix. IRIDA-ARIES is an open-source software that collects genomic and epidemiological data from the regional reference laboratories in order to get real-time data for timely detection of clusters of listeriosis and other foodborne diseases throughout Italy. For the analysis, the isolates of human origin belonging to the relevant ST were included.

A comparative analysis of cgMLSTs was carried out including all the public sequences of strains belonging to ST451, whose country of origin was ascertained. Sequences data and their information were retrieved from public databases such as Pasteur Institute BIGSdb-*Lm*, Ridom Gmbh, Germany (www.cgmlst.org/ncs/schema/690488/, accessed on 1 March 2021) and GenBank database (NCBI, www.ncbi.nlm.nih.gov/, accessed on 1 March 2021) (Table S3).

## Data Availability

Raw reads can be found in Sequence Read Archive (SRA) at GenBank database (NCBI, www.ncbi.nlm.nih.gov/, accessed on 1 March 2021) under the bioproject PRJNA708157, biosamples SAMN18220286, SAMN18220287, SAMN18220288, SAMN18220289, SAMN18220290, SAMN18631085, SAMN18631086, SAMN18631087, SAMN18631088, SAMN18631089. All the sequence data are available upon request to the corresponding author.

## Supplementary Materials

The following are available online at https://www.mdpi.com/article/10.3390/pathogens10050591/s1, Figure S1: Minimum spanning tree of all the strains consider in this study and others strains previously isolated from the same hospital; Figure S2: Minimum spanning tree of the available strains of ST451 in public databases; Table S1: SNPs distance matrix of every strains consider in this study; Table S2: List of virulence genes; Table S3: List of the public isolates used for the minimum spanning tree.
