# Supplementary material for: A Nosocomial Outbreak of Invasive Listeriosis in An Italian Hospital: Epidemiological and Genomic Features
Source: Pathogens. 2021 May 12;10(5):591. doi: 10.3390/pathogens10050591 (PMC8150339; doi:10.3390/pathogens10050591)
Supplement: Supplementary file 1 [file pathogens-10-00591-s001.zip › Supplementary Figure S1.pdf]

pathogens

MDPI

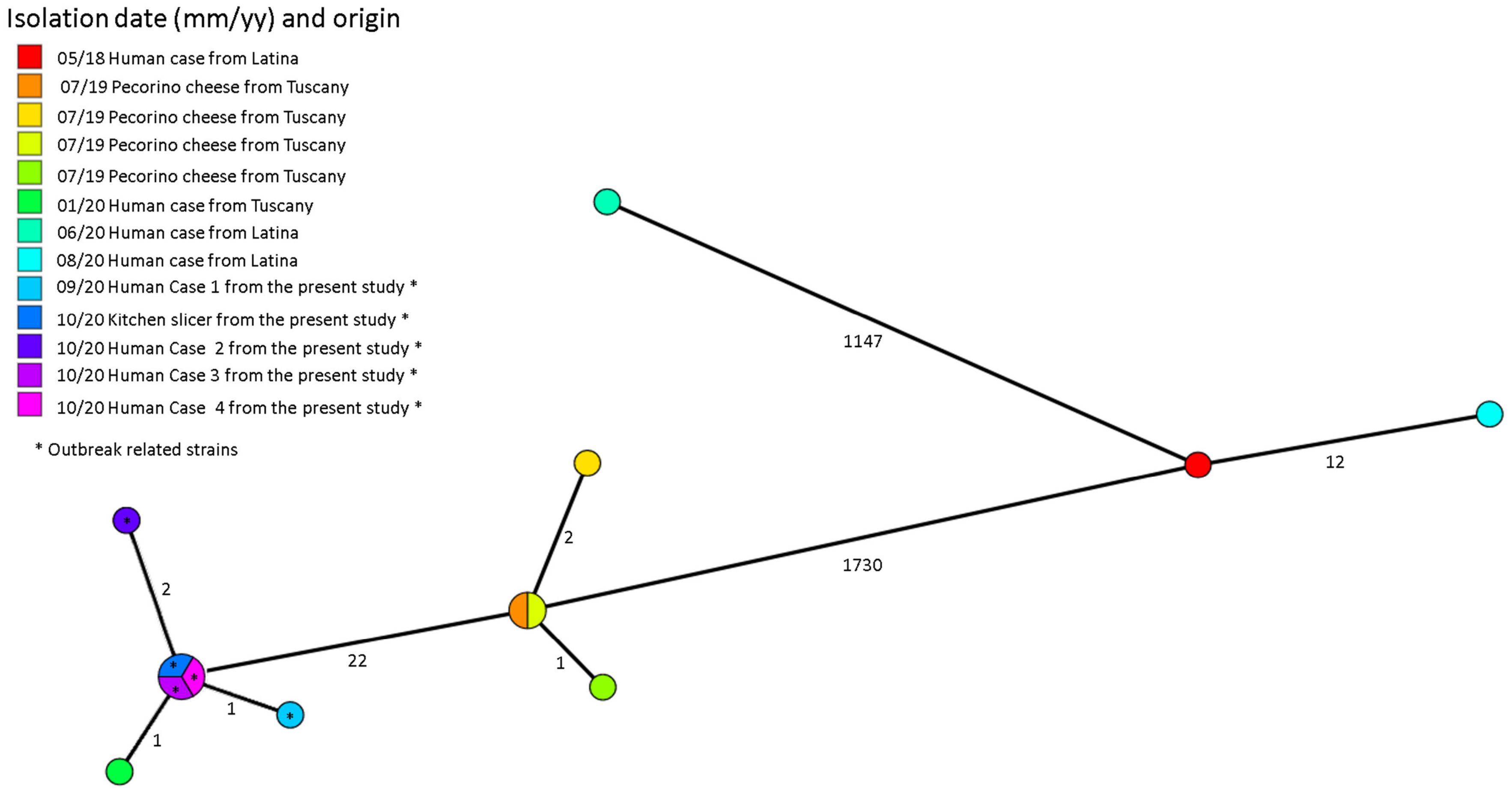

Supplementary Figure 1. Minimum Spanning Tree (MST) of all the human strains available to date from the Latina hospital, the ST451 strain from Tuscany, the isolate from the hospital kitchen slicer and the isolates from Pecorino cheese (Tuscany). For all the samples the month and year of isolation are indicated. The asterisks (*) indicate the strains correlated to the nosocomial outbreak described in the study.
