# Supplementary material for: A Nosocomial Outbreak of Invasive Listeriosis in An Italian Hospital: Epidemiological and Genomic Features
Source: Pathogens. 2021 May 12;10(5):591. doi: 10.3390/pathogens10050591 (PMC8150339; doi:10.3390/pathogens10050591)
Supplement: Supplementary file 1 [file pathogens-10-00591-s001.zip › Supplementary Figure S2.pdf]

pathogens

MDPI

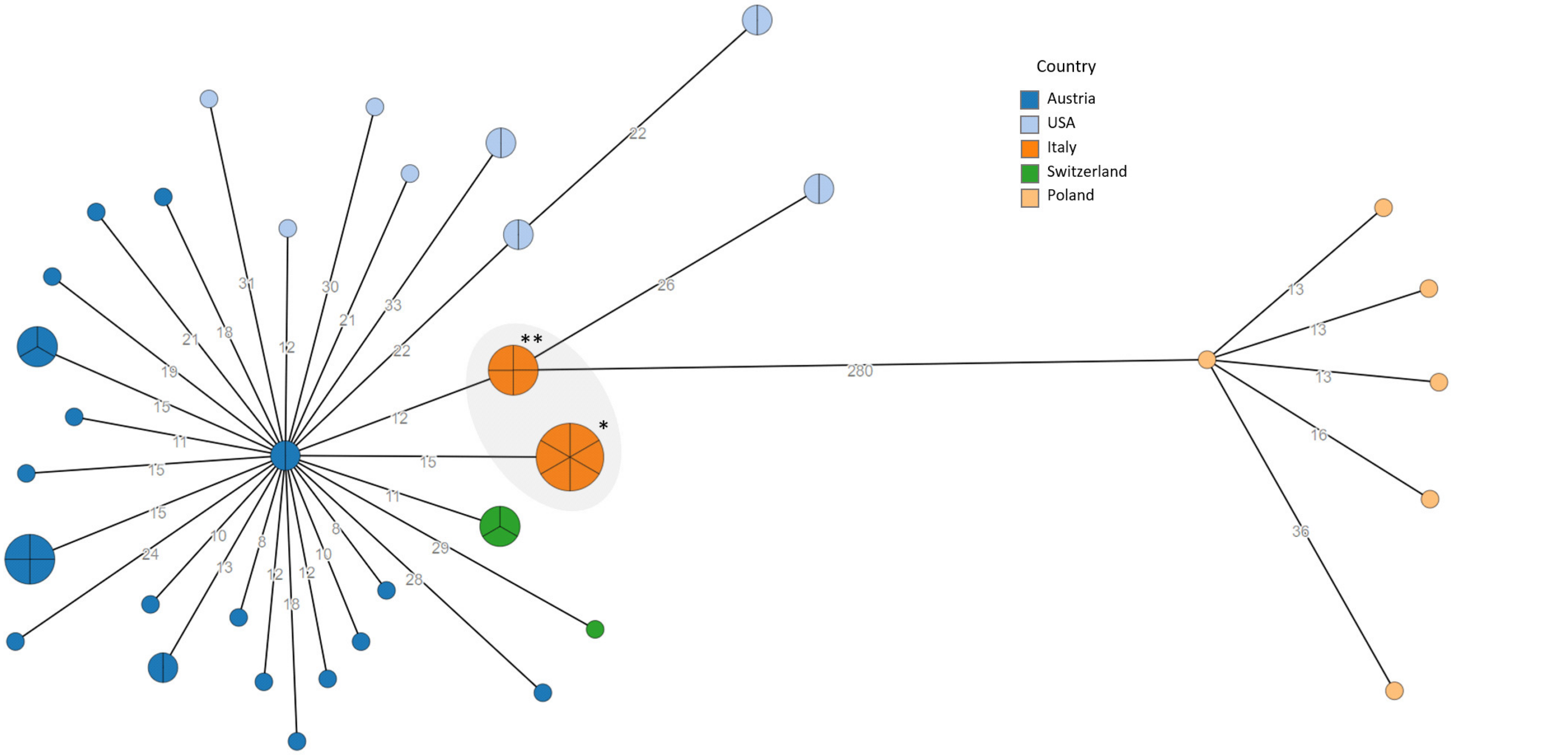

Supplementary Figure 2. Minimum Spanning Tree (MST) of all the ST451 strains with an ascertained origin available to date from public databases. The country of isolation for each sample is indicated in the legend. *: strains related to the nosocomial outbreak described in the study. **: strains of pecorino cheese.
